# Supplementary material for: Acceptance and compliance with micronutrient powder (MNP) among children aged 6–23 months in northern Nigeria
Source: PLOS Glob Public Health. 2022 Oct 17;2(10):e0000961. doi: 10.1371/journal.pgph.0000961 (PMC10022258; doi:10.1371/journal.pgph.0000961)
Supplement: S1 Dataset — (ZIP) [file pgph.0000961.s008.zip › S8_Dataset/In-depth Interviews.zip!/In-depth Interviews/110715_A_GU_BA_2_CG.pdf]

INDEPTH INTERVIEW (IDI) WITH WITH ABREME OF GUNDA COMMUNITY, GUYUK L.G.A ON 11/07/2015 IN ADAMAWA STATE.

My name is Abreme and I'm married with two kids. Presently we are four residing in this house and it includes my husband, the two kids and me. Presently, I have one daughter currently on MNP feeding and her name is Juliana and she's 22 months of age. My husband is a farmer and we equally assist him with the farm work. We do more of farming work during the raining season and we are in the busy season of the year and we have to work to keep the family going and ensure we don't lack food for the household. Food for young children is never a challenging because because they eat what we adult eat as well and we consume from what we cultivate at the farm. For children who are yet to cope with the nature of adult meal, they simply take the breast milk and soft food like kunu which are in varieties. The season affects the nature of food available in our community. Almost all the type of food we eat in our community is available throughout the season because we store our farm products. The major difference is that season affects how abundant and cheap such products are available. Example, maize is cheap in the raining season compared to other seasons. So, most households tend to consume more of maize product during that season. We cultivate maize and guinea corn in large quantity because our soil is good for such crop and kunu is a major food derived from such crops. This kunu is a major diet especially used as breakfast and it forms a major diet for children. We feed our children with kunu which come in varieties such as kunun dawa and kunun masara and kunun shinkafa. For our daughter on MNP feeding, we mix the MNP with kunu and she's mainly feed with it in the morning. The kunu is the only food we used to mix the MNP to feed her and I ensure it's given to her on daily basis. I've never missed a day without administering the MNP into her meal. For our children, we try to ensure they eat whatever we the adult eat and there is no special meal

prepared separately for them. My children and I eat varieties of food like tuwon dawa, wake, shinkafa and others and they like it.

There are various forms of tea in our community and people take them based on specific purposes. Some of the tea could be as food while others could be for medicinal purposes. We give our children tea made of milk, bournvita or milo and sugar in hot water to go along with bread. This meal is mainly taken in the morning as well and not on daily basis because it's much expensive and we have to manage the little we have. Whenever the tea is consumed as breakfast, the children will be feed with kunu about three to four hours after feeding with the tea and it will be an avenue to mix the kunu with MNP for the childwithin the age range. This feeding with kunu is ensured before lunch is ready. We mainly take this tea on Sundays which is not days for farming activities and the kunu is given to the children after we returned back from church and they needed something to eat before lunch is ready. My daughter on MNP does not take herbal medicines but I've given her once for stomach ache because the stomach pain was so severe and I tried other medication but to no avail. So I had o give it the herbal preparation a try. My experience with the herbal preration was that it made her passed lots of stool but there was relief but she lost weight and kept her weak for some days before full recovery.

Since I started giving her this MNP, she has added weight and increased in strength but there was diarrhea at the onset. Though the diarrhea lasted for the first few days when we started giving her the MNP, but it has subsided very well for now or I can say it has stopped because I can't remember when last she had the diarrhea. She's 22 month and no longer on breastfeeding but solely on house made foods such as kunu which the entire household feed on as well such as tuwon dawa and tuwon shinkafa.

We don’t have problem with storage of the MNP. I normally store it inside my bag and it’s well secured there and free from intruders including rodents. It was only once I tried to forget in giving her the MNP but I later gave her after coming back from church. The forgetfulness was not due to excess household jobs but trying to catch up with church services. One of the major causes of forgetfulness may be due to trying to catch up with important activities like farm work and so on. I have a question and not advice for people with forgetfulness. Please, what age range of children is meant to receive the MNP? Since my daughter is 22 months, it means one month is left for her to be legible for the MNP feeding. On the forgetfulness we were talking about earlier on, what is required is determination to use the MNP for the benefit of the child. Even if the mother is preparing other food, it’s just to get hot water from the pot on fire and prepare the kunu to be used with the MNP. It’s only kunu I used to mix the MNP.

Her eating habit has increased drastically since she started taking the MNP. Since I have 2 children and I have to avoid sharing, so, i used to fetch her kunu separately to include the MNP and try to avoid the attention of her elder brother. I used to dish a little of the kunu to mix with the MNP so that she can finish everything. Definitely that first quantity is not enough to satisfy her but just to make sure all the MNP from the sachet is consumed. After the first set of kunu, I then dish kunu for her without including the MNP so that if the kunu remains there won’t be problem on the MNP not used properly.

I was given two packet of MNP and each contains 30 sachets of MNP making a total of 60 sachets for having two packets. I’ll like to continue using the MNP if the 60 sachets finishes but its due to time and you said it’s meant for children below 23 months of age and she will soon be above the age range. If we continue to administer the MNP after she’s above the age

range, won't it cause any problem for her? The time is not a factor because I can give her at the right time without feeling any stressed but my concern is the age range she'll soon surpass. Kunu is not difficult to prepared, so if you said we should continue giving her, I won't hesitate provided its save for her.

I see the MNP as a drug because it increased her appetite for food and since she started taking the MNP, she doesn't have issue with fever and her teeth are now coming out properly and faster unlike then, they were coming out slowly and scanty. I'll be able and willing to pay for the MNP if it's to be sold because I've seen its functions which are good for my child's health. I can go to the drug store to purchase it for my child's use. We've been buying drugs like vitamin C to increase the appetite of children so that they can eat well which is needed to improve their health. Also, we've been buying other drugs to make the body of the child to be strong and healthy. If we are able to go to shops to buy those drugs, nothing will stop us from going out to buy this one because it has all the functions of other drugs we've used to improve the health of our baby. On amount I'll be willing to buy the MNP, I prefer little charges should be placed on the drug to make it affordable. As for me, I'll be able to pay 150 Naira per packet containing the 30 sachets inside. On ways to reach out to people about acceptance of the product, the hospital is a good channel. Mothers on antenatal or nursing their babies will hear about the product and start making on how to access the MNP or prepare money for future purchase of the drug if its to be sold. After hospital, other channels to reach out to people are through the church and village heads. These two channels are very reliable and efficient because the pastor and village heads are well respected and their words are trusted by every member of the community. I know how to prepare virtually all our traditional foods because while growing up as a girl, it's a major responsibility to learn cooking of local meals before marriage. But modernization has introduced some cusines

which are not part of our local meals and we have to learn them since our people have embraced the foods. So, on the foods I can't prepare, I simply meet people who can do so and I learn from them. Like the last time, I learnt how to bake chin chin from my pastor's wife. But it's majorly my pastors wife I learn some delicacies and outside her, there is no person specifically I've met on ideas about procedures for preparation of some foods.

Initially I didn't accept on MNP usage for my child when I was first approached but one of the distributor or staff of the programme later came to explain the importance of the product to me. After hearing him out, I saw some level of sincerity and its importance for my child. That conviction by her made me to accept the MNP and so far I have no regret but a big thanks to her for expressing such humility and time to explain what I lack knowledge about.

On places to disseminate MNP messages, example includes the church. You'll find large population of people there. I choose the church because it's a place of worship and people have much trust on the pastor. Most people attend church in my community, even those who didn't attend church service use to get feedback on the church sermon and general discussion at the service from friends and relative simply because people will like to implement what they have heard at the church service. So, those absent from the church service receive whatever information that was disseminated in church. The other avenue to disseminate information on the MNP is through the village square which is at the village head's compound. In that place, community meetings are held through the invitation of village head. The town crier moves round the whole community to announce for meeting with the village head and the subject of concern along with time for the meeting. People comprising of muslims, christains and those without religion will converge at the venue as announced and information will be disseminated and deliberations will go on till final verdict will be passed

by the head. Like if you are to come to introduce the MNP to create mass acceptance in my village and you want to use this method, the village head will stand up to introduce the message you have for the community at the gathering and you'll be giving the opportunity to give the full detail information of what you've brought to the community. After which deliberation will go on especially in a question and answer section and the village head will give the final verdict. If the verdict is favourable, the community will ensure a total cooperation to ensure you have an absolute success. Even among those who may disagree will comply because they will not want to go against the wish of the village head.

On issues of pass successful programmes of this nature in my community, I believe immunization against diseases such as poliomyelitis and measles is one of them. They equally passed through severe challenges before gaining acceptance. Up till date, some people (especially the fulanis) still reject vaccination against the disease. On their mode of operation, they distributed pamphlets or briefed the pastor on their intended activities. The pastor based on conviction of their motives and believed it is good and beneficial to her members then disseminated their message to the general congregation of the church in a manner that their programmes were accepted. From my observation, people accept totally with whatever advice or instructions given to them by the pastors.

Distribution of the MNP should be done by adopting the method of mosquitoe net distribution. The process involves the card system were households are given cards and after every distribution, the quantity supplied to various household will be registered on the card. Also, the pediatrics section of the hospital were nursing mothers take their children for immunization is another method. Since nursing mothers are already registered for immunization of their children at the hospital, the MNP should be combined into the

immunization exercise and it will be a success since immunization is already a success and widely accepted. In this method, whenever a nursing mother visits the hospital for immunization which has gained much publicity and acceptance, she will equally get the MNP. The house to house immunization was later strengthened because few people access the hospital due to money to pay hospital charges and distance to hospitals. On the house to house which you are currently doing, you are trying but it possess lot of challenges like gaining audience and people not willing to accept strangers into their home. Despite the presence of community members they are familiar with, people will not want to open up with strangers. Despite the challenges, I believe the house to house was a success with the immunization because population at the hospital was much and the staff never gave up. They pushed till the resilient once open their home for acceptance. Some had their children suffered the poliomyelitis before they learnt their lesson. My advice is to use all available methods to attain result so that if one fails, the other will succeed. A proverb in my village says, there are several ways to kill a rat and combined ways will improve your chance for success.

On the type of message to give to allow acceptance, I prefer the message to come during distribution and the content should cover the importance of the MNP to the health of the child both presently and in the future. Also the function of the MNP should be inculcated on the message on the message such as it prevents fever in children. Had it been I read the pamphlet given to me, I would have known the full functions of the MNP. Could you please explain in details the full functions of the MNP to the child because the only functions I can say now are those observed on the children since I started administering the MNP to her? Those functions observed in her included increase in weight and growth especially rapid growth of the teeth in my daughter due to the MNP.   Why I suggested that the messages for acceptance should

come during distribution of the MNP is because people tends to give their audience when there is something beneficial to receive. It’s a sign of motivation and it will be better during distribution to have the right words to go along for acceptance. On content of the messages to discourage sharing of the MNP among children, a thorough explanation should be given on why sharing should not be carried out. The message should be sharing will not profit both children i.e the one given the MNP and the other sharing it with him or her. It’ better for one of the child to take the full dosage as given while the other child wait till he or she get his or her own MNP. After all its possible to leave without the MNP and living without the MNP is not life threatening should be included on messages to discourage sharing. My suggestion is to carry out the distribution of the MNP on time to allow large scale access and use to influence positive change on the health and general wellbeing of the children.

On how to have continuity for the MNP program, it’s better for the government to support it in all possible areas because if you take a look at poliomyelitis, the government has a serious hand in it and it’s equally their major reason of attaining success in their activities. So my advice is for you to beg the government to be a major source of financial assistance to carry the burden if you are to succeed. I have much believe that the MNP project will succeed because you and your team are hard working to achieve your aim and the product has an exceptional importance for the children and I pray it succeed.

My question is when is the large scale distribution going to take place?

Hope my community will be among the first to benefit from such exercise when it happens?

I hope a proper channel for distribution will be adopted so that everyone will benefit and the procedure for collection will not be tedious?
